# Shift work is associated with extensively disordered sleep, especially when working nights

**DOI:** 10.3389/fpsyt.2023.1233640

**Published:** 2023-12-07

**Authors:** G. J. Boersma, T. Mijnster, P. Vantyghem, G. A. Kerkhof, Marike Lancel

**Affiliations:** ^1^Forensic Psychiatric Hospital, GGZ Drenthe Mental Health Institute, Assen, Netherlands; ^2^Centre of Expertise on Sleep and Psychiatry, GGZ Drenthe Mental Health Institute, Assen, Netherlands; ^3^Department of Clinical Psychology and Experimental Psychopathology, University of Groningen, Groningen, Netherlands; ^4^De Standaard (Daily Newspaper), Mediahuis, Brussels, Belgium; ^5^Department of Psychology, University of Amsterdam, Amsterdam, Netherlands; ^6^Sleep Disorders Center, Haaglanden Medical Center (HMC), The Hague, Netherlands

**Keywords:** work schedule, short/long sleep, sleep disorders, sleep disorder comorbidity, sociodemographic factors, education

## Abstract

**Background:**

Shift work is generally associated with working and sleeping out of phase with the endogenous, circadian sleep–wake cycle. This exerts detrimental effects on sleep health. The present study aimed at evaluating the presence of short and long sleep as well as sleep disorders within a broad range of shift work schedules and elucidating the role of sociodemographic factors therein.

**Methods:**

A large dataset containing information on sleep was collected through advertisement in a Belgium newspaper (De Standaard). Adult, working individuals were selected (n = 37,662) and categorized based on their work schedule (regular day, early morning, evening, night, and rotating shift). In this cross-sectional study, prevalence rates of short sleep (≤6 h), long sleep (≥9 h) and sleep disorders (screened with Holland Sleep Disorders Questionnaire), and associations between these sleep variables and sociodemographics (age, sex, education, living companion(s)) were analyzed using binominal logistic regression analyses.

**Results:**

In the total sample all sociodemographic factors affected prevalences of short, long and disordered sleep, consistent with previous studies. Compared to day workers, shift workers more frequently reported short sleep, most prominently night workers (26 vs. 50%) (*p* < 0.001). Furthermore, all sleep disorders as well as sleep disorder comorbidity were more common in shift workers, again most pronounced in night workers (all *p* < 0.05). In night shift workers the level of education had the strongest associations with disturbed sleep with a two-fold higher prevalence of short and disordered sleep in low relative to academic educated groups (all *p* < 0.02).

**Conclusion:**

Shift work is related not only to curtailed sleep and shift work disorder, but also to a plethora of sleep disorders, including insomnia, sleep-related breathing disorders and sleep-related movement disorders. Our findings imply that education on coping strategies may be especially important for young and/or lower educated shift workers.

## 1. Introduction

Sufficient and good quality sleep is critical to daytime functioning, physical and mental health. Lower sleep quality and/or shorter sleep duration are associated with diminished neurocognitive functions such as attention, memory and academic performance (1, 2). Both short sleep and poor sleep quality pose a risk for physical conditions like obesity, cardiovascular diseases and type 2 diabetes (3, 4). Though less often studied, long sleep duration is also associated with negative health outcomes, such as infectious and incident cardiovascular diseases (5, 6) and poorer cognitive functioning (7). With respect to mental health, disturbed sleep increases the risk for mood-, anxiety- and substance abuse-disorders, worsen daytime symptoms and impede remission of these mental diseases (8–11). In view of its overall importance, good sleep health is thus a major public health concern.

To assess sleep health, Buysse (12) proposed five quantifiable aspects of sleep, each clearly related to neurobehavioral, physical and mental well-being: sleep duration (total amount of sleep per 24 h), sleep efficiency (ease of falling and returning to sleep), sleep timing (sleep placement within the 24 h day), satisfaction with sleep (subjective sleep quality) and alertness (ability to maintain attentive wakefulness). A societal factor consistently interfering with sleep health is shift work, a situation where the behavioral sleep–wake schedule is out of phase with the endogenous, circadian sleep–wake rhythm and where additionally light exposure and feeding occur at non-optimal times of the day (13). A wealth of studies demonstrated that being forced to sleep in the biological day, during which the circadian system increasingly promotes wakefulness, is associated with problems falling and maintaining sleep and curtailed sleep (14–16). Concomitantly, having to work in the biological night, during which the circadian wake-promoting signal rapidly dissipates, is associated with higher levels of fatigue, sleepiness, performance impairments and accident-proneness (15, 17, 18). In about 27% of shift workers the sleep problems evolve into a shift work disorder (SWD) (19), a circadian rhythm sleep–wake disorder characterized by symptoms of insomnia, excessive sleepiness or both with a broad range of severe health consequences (20, 21). The significance of the disruptive effects of shift work is stressed by the fact that a considerable and rising number of people are working in shifts. For instance in 2010, 17% of all workers in the European Union were engaged in shift work and in 2015 the proportion had increased to 21% (22). Although underlying mechanisms have not been fully elucidated, the impact of shift work varies greatly between persons. This may be due to individual characteristics. For example, early chronotypes, so-called morning-types, appear sleepier during night shift hours than non-early chronotypes (23). Another example is the finding that tolerance to shift work differs between persons as a function of sleep quality (24). Variations in the impact of shiftwork may also be associated with work-related factors, such as permanent night shift workers sleeping consistently shorter than those in other shift types (25, 26). In spite of the large amount of studies on the effects of shift work on sleep quantity and quality, relatively little is known about the relation between work schedule and the presence of sleep disorders. A recent study found that a group of shift workers, compared to day workers, not only had a higher prevalence of SWD symptoms, but also of other, often comorbid, sleep disorders like insomnia, hypersomnia and sleep-related movement disorder, which was particularly strong for younger and single shift workers (27). Thus, shift work seems to provoke substantial sleep–wake disturbances.

The present study aimed to investigate the relation between a broad range of different shift work types (day, early morning, evening, night and rotating shift), the estimated presence of (co-occurring) sleep disorders and the influence of sociodemographic factors thereon. This study made use of a large dataset obtained from Belgian readers of a national newspaper who completed a screener for sleep disorders, the Holland Sleep Disorders Questionnaire (HSDQ), and provided sociodemographic information. Specifically, the estimated presence of short sleep duration (≤6 h), long sleep duration (≥9 h) and the six main groups of ICSD-defined sleep disorders (International Classification of Sleep Disorders, (28)) was assessed in the working population subdivided by sex, age, education level, living companions, and work schedule.

## 2. Methods

### 2.1. Procedure and study population

**Figure 1:**
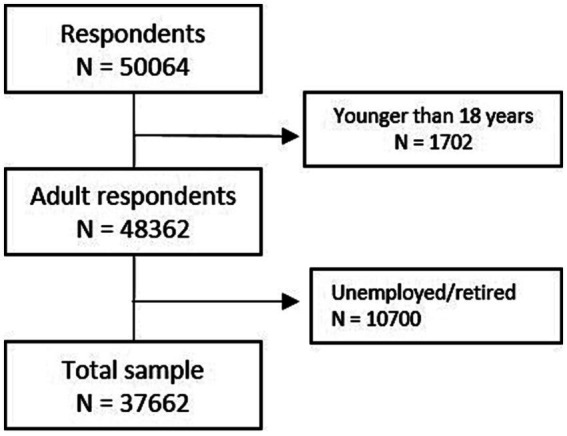
Flow chart representing the inclusion of participants in the study.

Participants were recruited via an advertisement on February 17th 2016 in a national Belgian newspaper, “De Standaard” (29). This newspaper is written in the Dutch language. The advertisement included a link to the online informed consent form and the questionnaires. The questionnaires consisted of demographic questions, the HSDQ, and some questions about sleep habits (bedtime, sleep duration). For the present study, respondents (n = 50,064) were only included if they were adult (≥18 years of age) and had a job working “regular day shift,” “regular early morning shift,” “regular evening shift,” “regular night shift” or “rotating shifts” (see Figure 1). The final sample consists of 37,662 participants and there were no missing data. This cross-sectional study presents point prevalence of short sleep duration, long sleep duration and six types of sleep disorders in the total sample and in subgroups based on demographic-factors. Additional analyses within the specific shift work groups into associations of the demographic variables were performed for three outcome variables known to be strongly affected by shift work, namely short sleep duration, presence of a sleep disorder and the presence of a circadian rhythm sleep wake disorder.

### 2.2. Demographic characteristics

Participants were asked to report their sex (male or female) and age. The age of the participants was categorized as younger than 30 years, between 30 and 50 years and older than 50 years. For the level of education participants could choose between elementary school, secondary school at lower level, secondary school at higher level, vocational training, university of applied sciences, and university. In the analyses, secondary school at low and higher level were grouped together as secondary education, and university of applied sciences and university as academic education. Participants were subdivided according to their work schedule (either regular day-, early morning-, evening-, night- or rotating-shifts). Concerning living companions, participants were asked to indicate whether they lived alone, with a partner, with children under the age of three, with children over three years old, with parents, with friend(s) and/or roommate(s). In the analyses, the living companions were grouped together in the following five categories: alone, with partner, alone with children, with partner and children, and with other(s).

### 2.3. Measurements

The Holland Sleep Disorders Questionnaire (HSDQ) is a 32-item questionnaire aimed at screening for the six main categories of the ICSD -defined (30) sleep disorders: insomnia, sleep-related breathing disorders (SBD), hypersomnia, parasomnia, circadian rhythm sleep–wake disorders (CRSWD), and sleep-related movement disorders (SRMD). Table 1 provides a brief description of these groups of sleep disorders. The HSDQ has been validated in a sample of the Dutch general population and has good psychometric properties (31). Items are scored on a 5-point Likert-scale (1 “never” to 5 “almost always”), whereby participants indicated to what extent an item applied to them in the last 3 months. For each participant the mean item score per sleep disorder subscale was calculated and compared to previously determined cut-off scores (insomnia: 3.68, SBD: 2.87, hypersomnia: 2.90, parasomnia: 2.42, CRSWD: 3.41, SRMD: 2.70). In addition, the total number of subscales scored above cut-off was used to calculate the suspected number of (comorbid) sleep disorders.

**Table 1:** Description of sleep disorder categories according to the ICSD-3.

Sleep disorder group	Brief description
Insomnia disorder	Persistent (≥3 months) and frequent (≥3 nights a week) difficulty initiating and/or maintaining sleep, resulting in daytime impairment.
Sleep breathing disorders (SBD)	Frequent (obstructive) respiratory events during sleep, associated with clinically relevant symptoms and/or diseases.
Hypersomnia	Excessive daytime sleepiness not explained by other sleep disorders.
Parasomnia	Undesirable behaviors or experiences during non-rapid eye movement (non-REM) sleep, REM sleep, or while transitioning between sleep and wake.
Circadian rhythm sleep–wake disorders (CRSWD)	Symptoms of insomnia and daytime impairment due to a misalignment between one’s endogenous sleep–wake rhythm and the external light–dark cycle.
Sleep-related movement disorders (SRMD)	Restless legs syndrome (RLS): unpleasant sensations in the legs paired with a strong urge to move the legs. Symptoms are worse at rest and during the evening/night and cause sleep disturbances and/or daytime impairments. Periodic Limb Movements during sleep (PLMD): involves sleep disruptive repetitive movements of the limbs.

Participants were asked to estimate the average duration they slept per 24 h. A binary variable, short sleep, was made differentiating those that slept six or less hours. A second binary variable was computed for long sleep, differentiating individuals sleeping nine of more hours.

### 2.4. Statistical analysis

Means and standard deviations for age and total sleep time were calculated. Statistical analyses were performed using SPSS software (IBM SPSS statistics 26). Distribution (% of the total population) of demographic and sleep parameters (presence of short, or long sleep duration and/or sleep disorders) were determined across the study sample. The prevalence with 95% confidence interval (CI = p ± 1.96*√((p*(1-p))/n), where p = point prevalence, and *n* = sample size) of sleep disorders as indicated by the HSDQ were assessed using cross-tables. Differences in the prevalence rates of sleep disorders between the different demographic categories were determined with chi-square tests. A Bonferroni correction was applied to the reported *p*-values to correct for multiple testing, and a value <0.05 was considered statistically significant. To express the size of the effects, prevalence ratio’s (PRs) (sample prevalence rate/ referent prevalent rate) were provided. Five subsamples were formed to compare and analyze the associations of sleep parameters and demographic factors within those working day (*n* = 32,468), early morning (*n* = 434), evening (*n* = 507), night (*n* = 186), and rotating (*n* = 4,067) shifts, respectively. In all subsamples, three binominal logistic regression analyses were performed with the presence of short sleep duration, a sleep disorder, and/or CRSWD as dependent variable, and sex, age, education, and living condition as independent variables. The male sex, an age below 30, an academic education and living alone were used as the indicator (referent) variables in these regression analyses. Alpha <0.05 was used to indicate statistical significance. PRs with 95% confidence intervals were calculated (e^LN(PR)^± 1.96 * √(1/a + 1/b + 1/c + 1/d), where PR = prevalence ratio, and a,b,c,d represent the cells of the cross-table) for each comparison.

## 3. Results

### 3.1. Study population

The average age of the final sample was 40.1 ± 12.0 years. The majority of the study population was female (59.4%), between 30 and 50 years old (49.0%), received an academic education (58.7%), performed daytime work (86.2%) and was living with partner and children (39.5%). On average the participants slept 6.97 ± 1.02 h per 24 h.

### 3.2. Prevalence of sleep disorders

About a quarter of the population reported short (≤6 h) sleep duration (Table 2; Supplementary Table S1.1) and about 5 percent long (≥9 h) sleep duration. The average sleep time for those with normal sleep duration was 7.46 ± 0.63 h, for those with short sleep duration 5.66 ± 0.63, and for those with a long sleep duration 9.00 ± 0.00 h. Approximately one third of the total sample scored positive on at least one sleep disorder subscale of the HSDQ, most frequently insomnia, closely followed by SRMD and CRSWD. Comorbid sleep disorders were also common; 12.6% screened positive for two or more sleep disorders.

**Table 2:** Percentage (95% CI) of the total sample with short sleep duration, long sleep duration and sleep disorders, differentiated by sex, age, education, living companion(s) and work schedule.

	≤6 h of sleep	≥9 h of sleep	≥1 sleep disorders	≥2 sleep disorders	Insomnia	SBD	Hypersomnia	Parasomnia	CRSWD	SRMD
Total population (*n* = 37,662)	27.5 (27.0–28.0)	5.3 (5.1–5.5)	33.3 (32.8–33.8)	12.6 (12.3–12.9)	12.8 (12.5–13.1)	5.9 (5.7–6.1)	6.4 (6.2–6.6)	5.1 (4.9–5.3)	9.4 (9.1–9.7)	12.6 (12.3–12.9)
*Sex*										
Males (*n* = 15,279)	30.9 (30.2–31.6)	4.0 (3.7–4.3)	30.4 (29.7–31.1)	11.0 (10.5–11.5)	10.1 (9.6–10.6)	8.3 (7.9–8.7)	4.8 (4.5–5.1)	3.4 (3.1–3.7)	8.7 (8.3–9.1)	11.5 (11.0–12.0)
Females (*n* = 22,383)	25.1 (24.5–25.7)	6.3 (6.0–6.6)	35.3 (34.7–35.9)	13.8 (13.3–14.3)	14.6 (14.1–15.1)	4.3 (4.0–4.6)	7.4 (7.1–7.7)	6.3 (6.0–6.6)	9.8 (9.4–10.2)	13.4 (13.0–13.8)
*Age*										
< 30 years (*n* = 9,146)	20.1 (19.3–20.9)	7.8 (7.3–8.3)	38.8 (37.8–39.8)	16.8 (16.0–17.6)	15.1 (14.4–15.8)	3.3 (2.9–3.7)	9.0 (8.4–9.6)	9.1 (8.5–9.7)	14.4 (13.7–15.1)	13.4 (12.7–14.1)
30–50 years (*n* = 18,450)	26.5 (25.9–27.1)	4.7 (4.4–5.0)	31.1 (30.4–31.8)	10.7 (10.3–11.1)	12.4 (11.9–12.9)	5.5 (5.2–5.8)	5.4 (5.1–5.7)	4.5 (4.2–4.8)	7.6 (7.2–8.0)	11.6 (11.1–12.1)
> 50 years (*n* = 10,066)	36.0 (35.1–36.9)	4.2 (3.8–4.6)	32.2 (31.3–33.1)	12.5 (11.9–13.1)	11.4 (10.8–12.0)	9.1 (8.5–9.7)	5.7 (5.2–6.2)	2.8 (2.5–3.1)	8.0 (7.5–8.5)	14.0 (13.3–14.7)
*Education*										
Academic (*n* = 22,119)	23.7 (23.1–24.3)	5.3 (4.9–5.5)	29.7 (29.1–30.3)	10.2 (9.8–10.6)	11.2 (10.8–11.6)	4.8 (4.5–5.1)	5.1 (4.8–5.4)	4.8 (4.5–5.1)	7.8 (7.4–8.2)	10.8 (10.4–11.2)
Vocational (*n* = 9,000)	29.6 (28.7–30.5)	5.1 (4.6–5.6)	34.7 (33.7–35.7)	13.4 (12.7–14.1)	13.8 (13.1–14.5)	6.5 (6.0–7.0)	6.8 (6.3–7.3)	4.9 (4.5–5.3)	9.3 (8.7–9.9)	13.3 (12.6–14.0)
Secondary (*n* = 6,401)	36.9 (35.7–38.1)	6.0 (5.4–6.6)	43.2 (42.0–44.4)	19.8 (18.8–20.8)	17.0 (16.1–17.9)	8.8 (8.1–9.5)	10.1 (9.4–10.8)	6.8 (6.2–7.4)	14.6 (13.7–15.5)	17.8 (16.9–18.7)
Elementary (*n* = 142)	46.5 (38.3–54.7)	7.0 (2.8–11.2)	52.8 (44.6–61.0)	22.5 (15.6–29.4)	18.3 (11.9–24.7)	15.5 (9.5–21.5)	10.6 (5.5–15.7)	8.5 (3.9–13.1)	18.3 (11.9–24.7)	22.5 (15.6–29.4)
*Living companion(s)*										
Alone (*n* = 4,705)	32.9 (31.6–34.2)	6.0 (5.3–6.7)	35.9 (34.5–37.3)	15.5 (14.5–16.5)	15.1 (14.1–16.1)	6.3 (5.6–7.0)	7.0 (6.3–7.7)	5.9 (5.2–6.6)	13.5 (12.5–14.5)	12.3 (11.4–13.2)
With partner (*n* = 10,663)	23.7 (22.9–24.5)	5.9 (5.5–6.3)	34.3 (33.4–35.2)	12.5 (11.9–13.1)	11.8 (11.2–12.4)	6.4 (5.9–6.9)	5.6 (5.2–6.0)	6.0 (5.5–6.5)	8.9 (8.4–9.4)	14.1 (13.4–14.8)
Alone with kid(s) (*n* = 2041)	35.3 (33.2–37.4)	4.1 (3.2–5.0)	33.7 (31.6–35.8)	12.2 (10.8–13.6)	13.4 (11.9–14.9)	6.9 (5.8–8.0)	7.3 (6.2–8.4)	4.3 (3.4–5.2)	8.0 (6.8–9.2)	12.1 (10.7–13.5)
With partner and kid(s) (*n* = 14,895)	28.1 (27.4–28.8)	3.9 (3.6–4.2)	29.2 (28.5–29.9)	9.8 (9.3–10.3)	11.3 (10.8–11.8)	6.0 (5.6–6.4)	5.5 (5.1–5.9)	3.2 (2.9–3.5)	6.0 (5.6–6.4)	11.5 (11.0–12.0)
Other(s) (*n* = 5,280)	25.2 (24.0–26.4)	8.1 (7.4–8.8)	40.3 (39.0–41.6)	18.5 (17.5–19.5)	16.8 (15.8–17.8)	4.1 (3.6–4.6)	9.5 (8.7–10.3)	8.5 (7.7–9.3)	16.5 (15.5–17.5)	13.7 (12.8–14.6)
*Work schedule*										
Day (*n* = 32,468)	26.3 (25.8–26.8)	5.0 (4.8–5.2)	31.5 (31.0–32.0)	11.4 (11.1–11.7)	12.3 (11.9–12.7)	5.6 (5.3–5.7)	5.5 (5.3–5.7)	4.9 (4.7–5.1)	7.8 (7.5–8.1)	12.1 (11.7–12.5)
Early (*n* = 434)	45.2 (40.5–49.9)	5.3 (3.2–7.4)	41.2 (36.6–45.8)	18.9 (15.2–22.6)	16.6 (13.1–20.1)	9.9 (7.1–12.7)	11.1 (8.1–14.1)	7.4 (4.9–9.9)	11.1 (8.1–14.1)	14.7 (11.4–18.0)
Evening (*n* = 507)	25.0 (21.2–28.8)	12.0 (9.2–14.8)	42.8 (38.5–47.1)	19.7 (16.2–23.2)	17.4 (14.1–20.7)	6.7 (4.5–8.9)	9.5 (6.9–12.1)	7.3 (5.0–9.6)	20.1 (16.6–23.6)	14.6 (11.5–17.7)
Night (*n* = 186)	49.5 (42.3–56.7)	7.0 (3.3–10.7)	51.1 (43.9–58.3)	26.4 (20.1–32.7)	19.9 (14.2–25.6)	10.2 (5.9–14.5)	15.6 (10.4–20.8)	8.1 (4.2–12.0)	24.7 (18.5–30.9)	22.6 (16.6–28.6)
Rotating (*n* = 4,067)	34.0 (32.5–35.5)	7.3 (6.5–8.1)	44.5 (43.0–46.0)	20.6 (19.4–21.8)	15.4 (14.3–16.5)	7.5 (6.7–8.3)	12.1 (11.1–13.1)	6.9 (6.1–7.7)	19.4 (18.2–20.6)	15.8 (14.7–16.9)

CRSWD = circadian rhythm sleep–wake disorder, SBD = sleep breathing disorder, SRMD = sleep-related movement disorder. Prevalence rates are provided in Supplementary Table S1.1.

A significantly larger proportion of males than females reported short sleep duration (*p* < 0.01), and a smaller proportion long sleep duration (*p* < 0.01). All sleep disorders were more common in females (insomnia *p* < 0.01; hypersomnia *p* < 0.01; parasomnia *p* < 0.01; CRSWD *p* < 0.05; SRMD *p* < 0.05), with the marked exception of SBD (*p* < 0.01). Comorbidity of sleep disorders was slightly higher in females (≥2 sleep disorders *p* < 0.01). With increasing age the prevalence of short sleep duration (*p* < 0.01) and SBD (*p* < 0.01) clearly increased. In contrast, long sleep duration (*p* < 0.01), insomnia (*p* < 0.05), hypersomnia (*p* < 0.01), parasomnia (*p* < 0.01) and CRSWD (*p* < 0.01), as well as sleep disorder comorbidity (*p* < 0.01) occurred most frequently in the youngest age group. Looking at education level, having a lower education (elementary or secondary) was associated with a significantly higher prevalence of both short (*p* < 0.01) and long (*p* < 0.05) sleep duration, all sleep disorder categories (insomnia *p* < 0.01; SBD *p* < 0.01; hypersomnia *p* < 0.01; parasomnia *p* < 0.05; CRSWD *p* < 0.01; SRMD *p* < 0.01) and comorbidity thereof (≥2 sleep disorders *p* < 0.01). Concerning living companions, living alone, living alone with children and/or with other(s) were generally related a higher occurrence of short sleep (*p* < 0.01) and all sleep disorders, except for parasomnia and SBD (insomnia p < 0.01; SBD p < 0.05; hypersomnia *p* < 0.01; parasomnia *p* < 0.01; CRSWD *p* < 0.01; SRMD *p* < 0.05). Also the proportion of people exhibiting comorbid sleep disorders (≥2 sleep disorders *p* < 0.01) was highest in these groups. In contrast, the prevalence of long sleep duration was highest in those living without children (*p* < 0.01). Regarding work schedules, about a quarter of the regular day and evening workers reported short sleep duration, yet the proportion was significantly higher (up to 49.5%) in those working early morning, night or rotating shifts (*p* < 0.01). Long sleep duration was more prevalent in those working evening shifts than in all other groups (*p* < 0.01). In day workers the most common sleep disorders were insomnia and SRMD, both occurred in about 12% of the population. All sleep disorders were more prevalent in those working on other work schedules, most strikingly insomnia, hypersomnia and CRSWD (insomnia *p* < 0.05; SBD *p* < 0.05; hypersomnia *p* < 0.01; parasomnia *p* < 0.05; CRSWD *p* < 0.01; SRMD *p* < 0.05). Of note, in the early morning shift CRSWD was only marginally increased, while all sleep disorders and sleep disorder comorbidity (≥2 sleep disorders *p* < 0.01) were most prevalent in the night shift workers; 51.1% of this group reported at least one sleep disorder and 26.3% at least two.

### 3.3. Short sleep duration within different work schedules

Independent of the type of work schedule, a larger percentage of men reported short sleep duration than women (Table 3; Supplementary Table S2.1). Although PRs were comparable, this effect only reached statistical significance in day, early morning and rotating shifts (day *p* < 0.05; early *p* < 0.05; evening *p* = 0.341; night *p* = 0.172; rotating *p* < 0.001). In general, increasing age was associated with a higher prevalence of short sleep (day *p* < 0.05; early *p* < 0.05; evening *p* < 0.05; night *p* = 0.168; rotating *p* < 0.001). The effect was slightly less pronounced in the early morning and night shifts (PR 1.26–1.31 vs. 1.7–1.9 in the other groups). For education, short sleep duration was more commonly reported by those with a lower education level in all work schedules (day *p* < 0.001; early *p* < 0.001; evening *p* < 0.05; night *p* < 0.05; rotating *p* < 0.001). In particular, those with only primary or secondary school significantly more often reported short sleep duration, with the PR being highest in early morning and night shifts. The influence of living companions is relatively small. Only in the day and rotation shift workers a significant association was found, with those living alone (with or without children) more frequently reporting short sleep than those living with a partner (day *p* < 0.001; early *p* = 0.494; evening *p* = 0.355; night *p* < 0.05; rotating *p* < 0.001).

**Table 3:** Prevalence and prevalence ratios of short (≤6 h) sleep duration in day, early morning, evening, night and rotating shift subsamples differentiated by sex, age, education and living companion (s).

	Day shift		Early shift		Evening shift		Night shift		Rotating shift	
	%	PR (95% CI)	%	PR (95% CI)	%	PR (95% CI)	%	PR (95% CI)	%	PR (95% CI)
*Sex*										
Male	29.5	1	51.2	1	27.0	1	57.1	1	38.7	1
Female	24.2	0.82 (0.77–0.87)**	39.6	0.77 (0.39–1.15)*	23.8	0.88 (0.47–1.29)	45.5	0.80 (0.18–1.41)	30.5	0.79 (0.66–0.92)**
*Age*										
< 30 years	18.7	1	41.3	1	20.1	1	39.1	1	26.3	1
30–50 years	25.4	1.36 (1.29–1.43)**	41.6	1.01 (0.52–1.49)	21.6	1.07 (0.56–1.59)	50.6	1.29 (0.36–2.23)	34.4	1.31 (1.15–1.47)**
> 50 years	34.7	1.86 (1.78–1.93)**	52.0	1.26 (0.76–1.76)	35.2	1.75 (1.23–2.28)*	51.4	1.31 (0.36–2.27)	43.3	1.65 (1.47–1.82)**
*Education*										
Academic	23.2	1	31.3	1	23.9	1	36.4	1	29.2	1
Vocational	28.7	1.24 (1.18–1.30)**	38.0	1.21 (0.65–1.78)	20.2	0.85 (0.26–1.43)	46.1	1.27 (0.50–2.03)	34.7	1.19 (1.02–1.35)*
Secondary	35.1	1.51 (1.45–1.58)**	56.6	1.81 (1.34–2.27)**	29.3	1.23 (0.75–1.71)*	62.9	1.73 (0.93–2.53)**	40.0	1.37 (1.22–1.52)**
Elementary ^$^	43.4	1.87 (1.47–2.27)**							48.0	1.64 (0.85–2.44)
*Living companion(s)*										
Alone	31.7	1	44.9	1	24.7	1	50.0	1	40.8	1
With partner	23.0	0.73 (0.64–0.81)**	40.3	0.89 (0.30–1.50)	22.6	0.91 (0.29–1.54)	42.9	0.86 (−0.10–1.81)	27.1	0.66 (0.45–0.88)
Alone with kid(s)^$^	33.9	1.07 (0.95–1.19)	52.2	1.16 (0.22–2.11)	40.7	1.65 (0.74–2.55)			44.8	1.10 (0.76–1.43)
With partner and kid(s)	26.9	0.85 (0.77–0.93)*	45.5	1.01 (0.44–1.59)	27.8	1.13 (0.53–1.72)	52.1	1.04 (0.15–1.94)	37.6	0.92 (0.72–1.12)
Other(s)	23.7	0.75 (0.65–0.84)**	50.0	1.11 (0.46–1.76)	20.4	0.83 (0.15–1.51)	47.8	0.96 (−0.17–2.08)	30.7	0.75 (0.53–0.97)

$ Low number of cases in the category of the early, evening and/or night shift subgroups (*n* < 20). *p < 0.05, ** *p* < 0.01. PR = prevalence ratio (calculated against indicator). Binominal logistic regression results in Supplementary Table S2.1.

### 3.4. Prevalence of any sleep disorder within different work schedules

The proportion of females having a sleep disorder generally exceeded that of men, with the exception of the early morning and evening shift (Table 4; Supplementary Table S2.2) (day *p* < 0.001; early *p* = 0.633; evening *p* = 0.785; night *p* = 0.065; rotating *p* < 0.05). Although prevalence rates were comparable in the day and night shift group, it did not reach statistical significance in the latter. Concerning age, in all work schedules those younger than 30 more frequently had a sleep disorder than the older age groups, with statistical significance in the day, evening and rotating shift workers (day *p* < 0.001; early *p* = 0.681; evening *p* < 0.05; night *p* = 0.715; rotating *p* < 0.05). In nearly all work schedules the rate of sleep disorders increased with lower education levels (significant in day, early, evening and rotating shifts) (day *p* < 0.001; early *p* < 0.05; evening *p* < 0.05; night *p* = 0.696; rotating *p* < 0.001). Merely in the night workers the prevalence of a sleep disorder was comparably high, independent of educational level. Finally, living alone or with other(s) generally tended to be associated with the highest occurrence of sleep disorders, although statistical significance was seldom reached (day *p* < 0.001; early *p* = 0.785; evening *p* = 0.155; night *p* = 0.656; rotating *p* < 0.01). More than half of the evening-, night- and rotating shift workers living alone or with others were estimated to suffer from disordered sleep.

**Table 4:** Prevalence and prevalence ratios of at least one sleep disorder in day, early, evening, night and rotating shift subsamples differentiated by sex, age, education and living companion (s).

	Day shift		Early shift		Evening shift		Night shift		Rotating shift	
	%	PR (95% CI)	%	PR (95% CI)	%	PR (95% CI)	%	PR (95% CI)	%	PR (95% CI)
*Sex*										
Male	28.4	1	42.5	1	42.9	1	46.0	1	42.3	1
Female	33.7	1.19 (1.14–1.23)**	40.1	0.94 (0.56–1.33)	42.8	1.00 (0.64–1.36)	53.7	1.17 (0.56–1.78)	46.0	1.09 (0.96–1.21)*
*Age*										
< 30 years	36.4	1	45.0	1	51.7	1	56.5	1	49.9	1
30–50 years	29.7	0.82 (0.76–0.87)**	41.0	0.91 (0.43–1.40)	42.7	0.83 (0.41–1.25)	47.2	0.84 (−0.09–1.76)	41.3	0.83 (0.68–0.97)*
> 50 years	30.7	0.84 (0.78–0.91)**	38.8	0.86 (0.36–1.36)	33.8	0.65 (0.18–1.12)*	54.1	0.96 (0.01–1.90)	42.6	0.85 (0.69–1.02)
*Education*										
Academic	28.5	1	36.7	1	37.0	1	50.0	1	40.5	1
Vocational	33	1.16 (1.10–1.21)**	32.6	0.89 (0.32–1.45)	50.6	1.37 (0.89–1.85)*	51.3	1.03 (0.28–1.77)	45.2	1.12 (0.96–1.27)
Secondary	41.3	1.45 (1.38–1.51)**	46.8	1.28 (0.82–1.73)	50.9	1.38 (0.94–1.81)*	48.4	0.97 (0.20–1.74)	49.3	1.22 (1.07–1.36)**
Elementary ^$^	48.5	1.70 (1.31–2.10)**							56.0	1.38 (0.59–2.18)**
*Living companion(s)*										
Alone	33.3	1	36.2	1	50.6	1	61.5	1	50.3	1
With partner	32.9	0.99 (0.91–1.07)	43.7	1.21 (0.60–1.82)	43.1	0.85 (0.32–1.39)	51.0	0.83 (−0.14–1.80)	42.6	0.85 (0.65–1.05)**
Alone with kid(s)^$^	32.5	0.98 (0.86–1.09)	39.1	1.08 (0.11–2.05)	44.4	0.88 (0.01–1.74)			42.5	0.84 (0.51–1.18)
With partner and kid(s)	27.9	0.84 (0.76–0.91)**	40.0	1.10 (0.51–1.70)	31.8	0.63 (0.09–1.17)*	47.9	0.78 (−0.13–1.69)	39.5	0.79 (0.59–0.98)**
Other(s)	37.7	1.13 (1.04–1.22)*	44.9	1.24 (0.58–1.90)	51.5	1.02 (0.45–1.59)	60.9	0.99 (−0.16–2.14)	50.8	1.01 (0.80–1.22)

$ Low number of cases in the category in the early, evening and/or night shift subgroups (*n* < 20). *p < 0.05, **p < 0.01. PR = prevalence ratio (calculated against indicator). Binominal logistic regression results in Supplementary Table S2.2.

### 3.5. Prevalence of a circadian rhythm sleep–wake disorder within different work schedules

Females more frequently scored on CRSWD than males in the regular day, early morning and night shift, with statistical significance being reached in the day shift (Table 5; Supplementary Table S2.3) (day *p* < 0.001; early *p* = 0.259; evening *p* = 0.296; night *p* = 0.210; rotating *p* = 0.587). In the rotating shift workers no sex difference was implied by the PRs. Rather independent of work schedule, CRSWD was most prevalent in the youngest age group (day *p* < 0.001; early *p* = 0.083; evening *p* < 0.05; night *p* = 0.227; rotating *p* < 0.01). Surprisingly, in the night workers no difference was observed between the youngest and oldest age groups. In the day workers, there was a strong effect of education level, with a lower education being associated with higher prevalence of CRSWD. The early, evening and rotating shift workers demonstrated a similar pattern, yet only significant in the rotating shift (day *p* < 0.001; early *p* = 0.180; evening *p* = 0.131; night *p* = 0.806; rotating *p* < 0.01). Within the night shift sample no effects of education were observed, with relatively high prevalence rates at all education levels. With the exception of the early morning shift, those living alone and with other(s) had the highest occurrence of CRSWD. These effects reached significance in the day, early, evening and rotating shifts (day *p* < 0.001; early *p* < 0.05; evening *p* < 0.05; night *p* = 0.084; rotating *p* < 0.001).

**Table 5:** Prevalence and prevalence ratios of a circadian rhythm sleep–wake disorder in day, early, evening, night and rotating shift subsamples differentiated by sex, age, education and living companion (s).

	Day shift		Early shift		Evening shift		Night shift		Rotating shift	
	%	PR (95% CI)	%	PR (95% CI)	%	PR (95% CI)	%	PR (95% CI)	%	PR (95% CI)
*Sex*										
Male	7.0	1	9.7	1	23.0	1	22.2	1	19.8	1
Female	8.4	1.20 (1.12–1.28)**	12.3	1.27 (0.66–1.88)	18.3	0.80 (0.36–1.24)	26.0	1.17 (0.45–1.89)	19.1	0.96 (0.81–1.12)
*Age*										
< 30 years	12.0	1	18.3	1	30.2	1	30.4	1	25.3	1
30–50 years	6.5	0.54 (0.45–0.64)**	5.2	0.28 (−0.54–1.11)	18.8	0.62 (0.13–1.11)	19.1	0.63 (−0.41–1.66)	16.3	0.64 (0.47–0.82)**
> 50 years	6.7	0.56 (0.45–0.67)**	12.5	0.68 (0.00–1.37)	11.7	0.39 (−0.23–1.00)**	29.7	0.98 (−0.04–2.00)	16.7	0.66 (0.46–0.86)**
*Education*										
Academic	6.8	1	8.6	1	16.8	1	27.3	1	16.9	1
Vocational	7.6	1.12 (1.02–1.22)**	4.3	0.50 (−0.68–1.68)	21.3	1.27 (0.68–1.86)	23.7	0.87 (0.02–1.72)	20.1	1.19 (0.99–1.39)**
Secondary	12.2	1.79 (1.69–1.90)**	15.1	1.76 (1.03–2.48)	26.7	1.59 (1.08–2.10)	22.6	0.83 (−0.06–1.72)	22.4	1.33 (1.14–1.51)**
Elementary	15.2	2.24 (1.68–2.79)**							20.0	1.18 (0.20–2.17)
*Living companion(s)*										
Alone	11.3	1	7.2	1	29.2	1	30.8	1	26.1	1
With partner	7.6	0.67 (0.55–0.80)**	8.4	1.17 (0.05–2.29)	18.2	0.62 (−0.01–2.25)*	20.4	0.66 (−0.42–1.75)	18.3	0.70 (0.46–0.94)**
Alone with kid(s)^$^	7.3	0.65 (0.44–0.85)**	8.7	1.21 (−0.51–2.92)	18.6	0.64 (−0.43–1.71)			13.3	0.51 (0.04–0.98)**
With partner and kid(s)	5.1	0.45 (0.33–0.58)**	6.9	0.96 (−0.16–2.07)	9.9	0.34 (−0.36–1.04)**	23.3	0.76 (−0.24–1.75)	13.6	0.52 (0.28–0.77)**
Other(s)	13.9	1.23 (1.10–1.36)	26.9	3.74 (2.70–4.78)*	30.1	1.03 (0.41–1.65)	39.1	1.27 (0.09–2.45)	26.3	1.01 (0.77–1.25)

$ Low number of cases in the category in the early, evening and/or night shift subgroups (*n* < 20). *p < 0.05, **p < 0.01. PR = prevalence ratio (calculated against indicator). Binominal logistic regression results in Supplementary Table S2.3.

## 4. Discussion

This study in a large sample of Belgian newspaper readers, confirmed previous findings (32) that there is a significant association between shift work and sleep problems. In the present study, this is expressed in both curtailed sleep duration and a high prevalence of estimated sleep disorders as well as sleep disorder comorbidity in all types of alternative work schedules, including early morning and evening shifts. The results implicate that regular night shift is by far the most debilitating condition at least concerning sleep; approximately half of the night workers reported short sleep (≤6 h), 51% scored positive on at least one sleep disorder, and 26% on two or more sleep disorders. An exception was long sleep duration, which was most prevalent in evening shift workers (12%). Several of the investigated sociodemographic factors influenced shift work-induced disordered sleep. The most prominent associations were found for the level of education; prevalence ratios of short sleep and one or more sleep disorders all increased about two-fold between the highest (academic) and lowest (elementary) education level.

### 4.1. Regular day shift

The influence of sociodemographic factors on sleep disturbances in the day workers followed similar patterns to those previously described in general populations. In both the current and earlier studies, being female was related to a higher risk of having a sleep disorder (except for SBD), while women less often reported short sleep duration (33–36). As is typically reported, short sleep becomes more common with increasing age (33–36). Yet consistent with previous findings, the prevalence of disordered sleep reduced with age, which was most prominently seen in the category of CRSWD (33, 36). Both sex and age were associated with short sleep duration and sleep disorders, but in opposite directions (being female and older is associated with lower short sleep, but higher sleep disorder occurrence). This underlines the notion that total sleep duration is one, but not the only aspect of healthy sleep (12).

Also consistent with previous findings, the level of education had a major impact on sleep, with both short sleep and sleep disorders being more common in lower educated individuals (37). The association with living-companionship was less straight forward across sleep measures, however, a general trend was that living alone (without partner and/or children) had negative consequences for sleep health. It is possible that those living alone receive less social pressure to maintain strict circadian patterns of behavior, which may impair their sleep hygiene practices. Alternatively, this group may receive less social (familial) support, which has been shown to reduce sleep health (38).

### 4.2. Regular night shift

The sample included a considerable group of fixed night shift workers (*n* = 186). A strikingly large proportion of this group reported short sleep duration (50%), and scored positive on the sleep disorders insomnia (20%), CRSWD (25%) and/or SRMD (23%). Compared to other types of shift work, night shift is associated with the largest change in sleep–wake timing. It is well-known that the circadian system resists the shift from a day- to a night-schedule and requires long lasting, consistent exposure to the altered sleep–wake and a matching light–dark schedule (21). It is estimated that merely 25% of the people adapt to night work (39). This is among others related to the fact that most night workers live in a day-oriented society (not in an isolated environment facilitating circadian adaptation), have domestic responsibilities, also during working days, and return to a relatively normal sleep–wake pattern on days off. Thus, particularly in fixed night workers there is an enduring, large misalignment between their endogenous and work-related sleep–wake schedule, negatively affecting sleep and many other health-related processes (21). The high prevalence of curtailed sleep and the different sleep disorders are in line with previous findings in shift workers [e.g., (25–27, 40)]. With regards to the prevalence of short sleep, insomnia and CRSWD, they are likely interrelated and might, at least partially, reflect the presence of SWD. That shift work-induced disordered sleep can be very persistent appears from a recent study demonstrating that night shift work is strongly associated with insomnia and daytime sleepiness even in the years after cessation of night work (41). The finding that also SRMD is rather common in night workers is in line with the literature [e.g., (27, 40)]. Underlying mechanisms are still unknown. There are implications that the SRMD restless legs syndrome (RLS) is associated with SWD. For instance, Waage and colleagues found in a sample of nurses that the prevalence of RLS was comparable in different shift work schedules, but was significantly higher in the nurses having SWD than in those without (42). The authors suggest that having one shift work-associated sleep disorder might increase the vulnerability to experience other sleep problems related to their work schedule. Alternatively, shift work is a risk factor for adverse mental health consequences, particularly depressive symptoms. Meta-analyses demonstrated a 33% increase for depressive symptoms among shift workers (43) and a 42% increase for depression in night workers (44). Various antidepressants trigger and aggravate SRMD (45). Yet, studies on the use of psychiatric medication in shift workers are inconclusive (46, 47).

In contrast to earlier studies (27, 40), we found a significant doubling of estimated SBD in the night compared to day workers. At this point, an explanation for this discrepancy remains elusive. Yet, a polysomnographic study in shift workers with obstructive sleep apnea syndrome found that the apnea-hypopnea index (AHI) is significantly higher during sleep scheduled in the day time than during night time (48). The increase in obstructive sleep apnea severity may be related to the high occurrence of respiratory events during rapid eye movement (REM) sleep, a sleep state that occurs most during the biological morning, a time of day during which night shift workers are typically sleeping (49).

Within night workers, sex, age and living companions had a relatively minor association with the already very prevalent short sleep duration. Especially the absence of differences between the age groups might be considered unexpected, as several studies report more sleep problems in older night shift workers compared to younger workers [e.g., (50–52)]. However, consistent with our data a recent meta-analysis on sleep quality revealed a moderating effect of age in those working rotation shifts, but an absence hereof in the night shift workers (53). In contrast, the level of education had a significant impact with a PR of 1.7. This might be related to differences in the type of profession between those with a high and a low education level (e.g., factory work vs. working as a physician). Furthermore, education level is often used as a proxy for socioeconomic status, and thus may be tied to the living conditions, for example living in a small, noisy apartment versus independent housing. The ability to adapt to the challenging circumstances may also hamper sleep. Concerning the presence of any sleep disorders, none of the sociodemographic factors was a significantly associated with this outcome parameter. Considering the overall high prevalence of sleep disorders in regular night workers, this may be explained by a ceiling effect. This notion is strengthened by the fact that prevalence rates were higher in evening and rotating shifts to the level, but not beyond, of night workers as a function of sociodemographic factors.

### 4.3. Early morning work

Rather independent of sociodemographic factors, working early mornings had little effect on the occurrence of sleep disorders, including CRSWD. However, it was associated with a high prevalence of short sleep duration. This higher prevalence of curtailed sleep may be explained by 1) going to bed earlier, experiencing problems initiating sleep (21), and 2) keeping normal bedtimes, while rising earlier on work days. The contradiction between an association with short sleep and the absence thereof with most sleep disorders may be due to early workers maintaining relatively normal night and day rhythms, which may protect them from circadian misalignment and sleep disorders. The only sleep disorder that was clearly more prevalent in early morning workers was hypersomnia, with a 2-fold PR compared to day workers. Restricted sleep has been shown to induce accumulating levels of sleepiness during the day in both naturalistic and experimental settings (54, 55). Thus, the higher risk to hypersomnia might result from the repeated shortened sleep duration in early morning workers. Of note, the relationship of an early morning shift with short sleep seems weaker in females than in males. This difference is possibly explained by women more often being early birds (56), thus requiring less adaptation to an early morning shift. In the early morning workers, the prevalence of CRSWD was strikingly higher (3.7 fold) in the “others” living companion group. Within the ‘others’ group CRSWD estimates were high in those living with their parents (27% with CRSWD), whereas those living with friends (11% with CRSWD) displayed prevalences more similar to early morning workers living alone, with partner and/or children. In our sample, individuals living with their parents had lower education levels than those living with friends (80% vs. 33% with only primary or secondary education), which may partially explain the heightened prevalence of CRSWD in this group.

### 4.4. Evening and rotating shifts

Overall the associations within evening and rotating shifts on sleep are less pronounced, but comparable to those of night shift workers. Furthermore, the relationships of sociodemographic factors with sleep parameters followed similar patterns as in day shifts, with younger, lower educated individuals, living without partner and kid(s), being at highest risk for disordered sleep. Shift work is indicated to be associated with a slight increase in the prevalence of long sleep duration (6). In the present study, this association was only significant for the evening shift. It is conceivable that evening workers tend to go to bed soon after work, at a biological time of high sleep propensity, and sleep in as they do not need to get up early for work.

Unfortunately, in the current study we were not able to differentiate between different orders of shifts within the rotating shift group. Previous studies have shown that those working in a backward rotating shift schedule tend to have more sleep problems than those working in a forward schedule (57, 58). Given the known effects of the order of shifts, future studies should take this into account.

### 4.5. Limitations

There are some limitations to this study, the first concerning the study sample. Due to the recruitment of participants through a newspaper advertisement, a selection bias is likely. For example, compared to the general Belgian population (59), the current sample was clearly higher educated (14.6% with academic education in general population in 2016 versus 59% in current sample). Furthermore, individuals with sleep problems might be more inclined to participate in a study focused on sleep than good sleepers, and thus persons with sleep disturbances may be overrepresented in our sample. This may be evidenced by the presently observed slightly higher prevalence of sleep disturbances in comparison with a study in a representative sample of the Dutch population (33). However, differences between the countries may play a role here as well, with previous reports of more self-reported sleep problems in Belgium than in the Netherlands (60). Nevertheless, although absolute prevalence rates might be overestimated in our study, we believe it unlikely that the associations with the investigated sociodemographic factors are strongly affected by such a selection bias.

A second limitation is the use of a screenings-questionnaire to assess the prevalence of sleep disorders. Though the used HSDQ has good clinical validity (31), a questionnaire can only provide an estimate. Especially those sleep disorders that require polysomnography for diagnosis, like SBD, are less accurately assessed using a questionnaire.

Finally, using a naturalistic study design has led to large differences in the sample sizes of the work shift subgroups. This complicated direct comparisons between subsamples as similar prevalence ratios reached statistical significance in the larger subsamples, yet failed to do so in the smaller subsamples.

### 4.6. Implications and perspectives

Compared to regular day work, all shift work schedules seem associated with adverse sleep effects; night shifts most strongly. As societies cannot do without night work and adaptation to it is hard and often undesirable, rotational shifts are generally advised, particularly fast-forward rotating work schedules, with the period of night work as short as possible and plenty of resting days in between to recover from the accumulated sleep deficit (61, 62). To prevent sleep curtailment and sleep disorders, employers/occupational health practitioners should encourage good sleep health and give tools to deal with shift work as well as possible, both promoting optimal sleep during the resting period and wakefulness during working hours. It would, for instance, be useful to educate shift workers, and ideally their family members and/or other supporting living companions, on homeostatic and circadian regulation of sleep, good sleep hygiene (avoiding alcohol and caffeine close to bedtime), and optimization of the sleep environment (dark, cool and quiet bedroom), and reduction of sleepiness during the working period by for instance powernaps, caffeine and bright light. Such information could be particularly helpful for young and low educated shift workers, as these groups appear to experience most sleep problems and may benefit most from information on coping strategies. Furthermore, regular assessment of sleep quality and quantity and screening for disordered sleep in those working shifts might be crucial to timely treat sleep disorders such as SWD and insomnia, and thereby preventing persistent sleep disturbances and their adverse effects on physical and mental health and work performance.

## Data availability statement

The raw data supporting the conclusions of this article will be made available by the authors, without undue reservation.

## Ethics statement

Ethical approval was not required for the studies involving humans because The study involved a questionnaire published in a newspaper. Participants could freely choose whether to complete the questionnaire or not. The studies were conducted in accordance with the local legislation and institutional requirements. The participants provided their written informed consent to participate in this study.

## Author contributions

PV and GK were involved in data collection. GB, TM, and ML were involved in data analysis. GB and ML were responsible for conceptualization and the first draft of the manuscript. All authors reviewed and provided feedback on the final manuscript. All authors contributed to the article and approved the submitted version.

## References

[1] Hudson AN Van Dongen HP Honn KA. Sleep deprivation, vigilant attention, and brain function: a review. Neuropsychopharmacology. (2020) 45:21–30. doi: 10.1038/s41386-019-0432-6, PMID: 31176308 PMC6879580

[2] Okano K Kaczmarzyk JR Dave N Gabrieli JD Grossman JC. Sleep quality, duration, and consistency are associated with better academic performance in college students. NPJ Science Learn. (2019) 4:16. doi: 10.1038/s41539-019-0055-z PMC6773696 31583118

[3] Cappuccio FP Miller MA. Sleep and cardio-metabolic disease. Curr Cardiol Rep. (2017) 19:1–9. doi: 10.1007/s11886-017-0916-0 28929340 PMC5605599

[4] Reutrakul S Van Cauter E. Sleep influences on obesity, insulin resistance, and risk of type 2 diabetes. Metab Clin Exp. (2018) 84:56–66. doi: 10.1016/j.metabol.2018.02.010, PMID: 29510179

[5] Jike M Itani O Watanabe N Buysse DJ Kaneita Y. Long sleep duration and health outcomes: a systematic review, meta-analysis and meta-regression. Sleep Med Rev. (2018) 39:25–36. doi: 10.1016/j.smrv.2017.06.011, PMID: 28890167

[6] Prather AA Carroll JE. Associations between sleep duration, shift work, and infectious illness in the United States: data from the National Health Interview Survey. Sleep Health. (2021) 7:638–43. doi: 10.1016/j.sleh.2021.05.004, PMID: 34193397

[7] Lo JC Groeger JA Cheng GH Dijk DJ Chee MW. Self-reported sleep duration and cognitive performance in older adults: a systematic review and meta-analysis. Sleep Med. (2016) 17:87–98. doi: 10.1016/j.sleep.2015.08.021, PMID: 26847980

[8] Riemann D Krone LB Wulff K Nissen C. Sleep, insomnia, and depression. Neuropsychopharmacology. (2020) 45:74–89. doi: 10.1038/s41386-019-0411-y, PMID: 31071719 PMC6879516

[9] Belleville G Cousineau H Levrier K St-Pierre-Delorme M. Meta-analytic review of the impact of cognitive-behavior therapy for insomnia on concomitant anxiety. Clin Psychol Rev. (2011) 31:638–52. doi: 10.1016/j.cpr.2011.02.004, PMID: 21482322

[10] Lancel M van Marle HJ Van Veen MM van Schagen AM. Disturbed sleep in PTSD: thinking beyond nightmares. Front Psych. (2021) 12:2135. doi: 10.3389/fpsyt.2021.767760 PMC8654347 34899428

[11] Roehrs T Sibai M Roth T. Sleep and alertness disturbance and substance use disorders: a bi-directional relation. Pharmacol Biochem Behav. (2021) 203:173153. doi: 10.1016/j.pbb.2021.173153, PMID: 33582097 PMC7996967

[12] Buysse DJ. Sleep health: can we define it? Does it matter? Sleep. (2014) 37:9–17. doi: 10.5665/sleep.3298, PMID: 24470692 PMC3902880

[13] Meyer N Harvey AG Lockley SW Dijk D. Circadian rhythms and disorders of the timing of sleep. Lancet. (2022) 400:1061–78. doi: 10.1016/S0140-6736(22)00877-7 36115370

[14] Åkerstedt T. Shift work and disturbed sleep/wakefulness. Occup Med. (2003) 53:89–94. doi: 10.1093/occmed/kqg046, PMID: 12637592

[15] Drake CL Roehrs T Richardson G Walsh JK Roth T. Shift work sleep disorder: prevalence and consequences beyond that of symptomatic day workers. Sleep. (2004) 27:1453–62. doi: 10.1093/sleep/27.8.1453, PMID: 15683134

[16] Fossum IN Bjorvatn B Waage S Pallesen S. Effects of shift and night work in the offshore petroleum industry: a systematic review. Ind Health. (2013) 51:530–44. doi: 10.2486/indhealth.2013-0054, PMID: 23803497 PMC4202738

[17] Folkard S Lombardi DA Tucker PT. Shiftwork: safety, sleepiness and sleep. Ind Health. (2005) 43:20–3. doi: 10.2486/indhealth.43.20 15732299

[18] Åkerstedt T Wright KP. Sleep loss and fatigue in shift work and shift work disorder. Sleep Med Clin. (2009) 4:257–71. doi: 10.1016/j.jsmc.2009.03.001, PMID: 20640236 PMC2904525

[19] Pallesen S Bjorvatn B Waage S Harris A Sagoe D. Prevalence of shift work disorder: a systematic review and meta-analysis. Front Psychol. (2021) 12:638252. doi: 10.3389/fpsyg.2021.638252, PMID: 33833721 PMC8021760

[20] Wickwire EM Geiger-Brown J Scharf SM Drake CL. Shift work and shift work sleep disorder: clinical and organizational perspectives. Chest. (2017) 151:1156–72. doi: 10.1016/j.chest.2016.12.007, PMID: 28012806 PMC6859247

[21] Boivin DB Boudreau P Kosmadopoulos A. Disturbance of the circadian system in shift work and its health impact. J Biol Rhythm. (2022) 37:3–28. doi: 10.1177/07487304211064218, PMID: 34969316 PMC8832572

[22] Parent-Thirion A Biletta I Cabrita J Llave OV Vermeylen G Wilczynska A. 6th European working conditions survey: 2017 update. Luxembourg: Publications Office of the European Union (2017).

[23] Hilliker NAJ Muehlbach MJ Schweitzer PK Walsh JK. Sleepiness/alertness on a simulated night shift schedule and morningness–eveningness tendency. Sleep. (1992) 15:430–3. doi: 10.1093/sleep/15.5.430, PMID: 1455126

[24] Lammers-van der Holst HM Kerkhof GA. Shift work tolerance and the importance of sleep quality: a study of police officers. Biol Rhythm Res. (2015) 46:257–64. doi: 10.1080/09291016.2014.985002

[25] Ohayon MM Lemoine P Arnaud-Briant V Dreyfus M. Prevalence and consequences of sleep disorders in a shift worker population. J Psychosom Res. (2002) 53:577–83. doi: 10.1016/S0022-3999(02)00438-5 12127174

[26] Cheng W Cheng Y. Night shift and rotating shift in association with sleep problems, burnout and minor mental disorder in male and female employees. Occup Environ Med. (2017) 74:483–8. doi: 10.1136/oemed-2016-103898 27810939

[27] Kerkhof GA. Shift work and sleep disorder comorbidity tend to go hand in hand. Chronobiol Int. (2018) 35:219–28. doi: 10.1080/07420528.2017.1392552, PMID: 29157012

[28] American Academy of Sleep Medicine. The international classification of sleep disorders. 3rd ed. Darien: American Academy of Sleep Medicine (2014)).

[29] Mediahuis (2023) Available at: https://www.standaard.be (accessed May 2023).

[30] American Academy of Sleep Medicine. The international classification of sleep disorders. 2nd ed (2004).

[31] Kerkhof GA Geuke ME Brouwer A Rijsman RM Schimsheimer RJ Van Kasteel V. Holland sleep disorders questionnaire: a new sleep disorders questionnaire based on the international classification of sleep Disorders-2. J Sleep Res. (2013) 22:104–7. doi: 10.1111/j.1365-2869.2012.01041.x 22924964

[32] Hernandez AFJ Bautista RLS Tan CC. Sleep disturbances during shift work. Sleep Med Clin. (2022) 17:1–10. doi: 10.1016/j.jsmc.2021.10.001 35216756

[33] Kerkhof GA. Epidemiology of sleep and sleep disorders in the Netherlands. Sleep Med. (2017) 30:229–39. doi: 10.1016/j.sleep.2016.09.015 28215254

[34] Kocevska D Lysen TS Dotinga A Koopman-Verhoeff ME Luijk MP Antypa N. Sleep characteristics across the lifespan in 1.1 million people from the Netherlands, United Kingdom and United States: a systematic review and meta-analysis. Nat Hum Behav. (2021) 5:113–22. doi: 10.1038/s41562-020-00965-x, PMID: 33199855

[35] Kronholm E Härmä M Hublin C Aro AR Partonen T. Self-reported sleep duration in Finnish general population. J Sleep Res. (2006) 15:276–90. doi: 10.1111/j.1365-2869.2006.00543.x, PMID: 16911030

[36] Ram S Seirawan H Kumar SK Clark GT. Prevalence and impact of sleep disorders and sleep habits in the United States. Sleep Breath. (2010) 14:63–70. doi: 10.1007/s11325-009-0281-3 19629554

[37] Etindele Sosso FA Kreidlmayer M Pearson D Bendaoud I. Towards a socioeconomic model of sleep health among the Canadian population: a systematic review of the relationship between age, income, employment, education, social class, socioeconomic status and sleep disparities. Euro J Invest Health Psychol Educn. (2022) 12:1143–67. doi: 10.3390/ejihpe12080080, PMID: 36005229 PMC9407487

[38] Kent de Grey RG Uchino BN Trettevik R Cronan S Hogan JN. Social support and sleep: a meta-analysis. Health Psychol. (2018) 37:787–98. doi: 10.1037/hea0000628 29809022

[39] Folkard S. Do permanent night workers show circadian adjustment? A review based on the endogenous melatonin rhythm. Chronobiol Int. (2008) 25:215–24. doi: 10.1080/07420520802106835, PMID: 18533325

[40] Garbarino S De Carli F Nobili L Mascialino B Squarcia S Penco MA. Sleepiness and sleep disorders in shift workers: a study on a group of Italian police officers. Sleep. (2002) 25:642–7. 12224843

[41] Weitzer J Santonja I Degenfellner J Yang L Jordakieva G Crevenna R. Sleep complaints in former and current night shift workers: findings from two cross-sectional studies in Austria. Chronobiol Int. (2021) 38:893–06. doi: 10.1080/07420528.2021.1895200, PMID: 33757396

[42] Waage S Pallesen S Moen BE Bjornvatn B. Restless legs syndrome/Willis-Ekbom disease is prevalent in working nurses, but seems not to be associated with shift work schedules. Front Neurol. (2018) 9:21. doi: 10.3389/fneur.2018.00021 29434568 PMC5796891

[43] Torquati L Mielke GI Brown WJ Burton NW Kolbe-Alexander TL. Shift work and poor mental health: a meta-analysis of longitudinal studies. Am J Public Health. (2019) 109:e13–20. doi: 10.2105/AJPH.2019.305278, PMID: 31536404 PMC6775929

[44] Angerer P Schmook R Elfantel I Li J. Night work and the risk of depression: a systematic review. Dtsch Arztebl Int. (2017) 114:404–11. doi: 10.3238/arztebl.2017.0404, PMID: 28669378 PMC5499504

[45] Ferri R Mogavero MP Bruni O Picchietti DL LM DR. Periodic leg movements during sleep associated with antidepressants: a meta-analysi. Neurosci Biobehav Rev. (2023) 148:105126. doi: 10.1016/j.neubiorev.2023.105126 36914081

[46] Laaksonen M Lallukka T Lahelma E Partonen T. Working conditions and psychotropic medication: a prospective cohort study. Soc Psychiatry Psychiatr Epidemiol. (2012) 47:663–70. doi: 10.1007/s00127-011-0372-x 21445624

[47] Hall AL Kecklund G Leineweber C Tucker P. Effect of work schedule on prospective antidepressant prescriptions in Sweden: a 2-year sex-stratified analysis using national drug registry data. BMJ Open. (2019) 9:e023247. doi: 10.1136/bmjopen-2018-023247, PMID: 30782699 PMC6340477

[48] Paciorek M Korczyński P Bielicki P Byśkiniewicz K Zieliński J Chazan R. Obstructive sleep apnea in shift workers. Sleep Med. (2011) 12:274–7. doi: 10.1016/j.sleep.2010.06.013 21316298

[49] Reid KJ Abbott SM. Jet lag and shift work disorder. Sleep Med Clin. (2015) 10:523–35. doi: 10.1016/j.jsmc.2015.08.006 26568127

[50] Härmä M. Ageing, physical fitness and shiftwork tolerance. Appl Ergon. (1996) 27:25–9. doi: 10.1016/0003-6870(95)00046-1 15676308

[51] Bonnefond A Härmä M Hakola T Sallinen M Kandolin I Virkkala J. Interaction of age with shift-related sleep-wakefulness, sleepiness, performance, and social life. Exp Aging Res. (2006) 32:185–08. doi: 10.1080/03610730600553968, PMID: 16531360

[52] Seo YJ Matsumoto K Park YM Shinkoda H Noh TJ. The relationship between sleep and shift system, age and chronotype in shift workers. Biol Rhythm Res. (2000) 31:559–79. doi: 10.1076/brhm.31.5.559.5655

[53] Chang WP Peng YX. Meta-analysis of differences in sleep quality based on actigraphs between day and night shift workers and the moderating effect of age. J Occup Health. (2021) 63:e12262. doi: 10.1002/1348-9585.12262, PMID: 34392580 PMC8364763

[54] Bartlett DJ Marshall NS Williams A Grunstein RR. Sleep health New South Wales: chronic sleep restriction and daytime sleepiness. Intern Med J. (2008) 38:24–31. doi: 10.1111/j.1445-5994.2007.01395.x, PMID: 17543000

[55] Van Dongen HP Maislin G Mullington JM Dinges DF. The cumulative cost of additional wakefulness: dose-response effects on neurobehavioral functions and sleep physiology from chronic sleep restriction and total sleep deprivation. Sleep. (2003) 26:117–26. doi: 10.1093/sleep/26.2.117, PMID: 12683469

[56] Randler C. Gender differences in morningness–eveningness assessed by self-report questionnaires: a meta-analysis. Personal Individ Differ. (2007) 43:1667–75. doi: 10.1016/j.paid.2007.05.004

[57] Van Amelsvoort LG Jansen NW Swaen GM Van Den Brandt PA Kant I. Direction of shift rotation among three-shift workers in relation to psychological health and work-family conflict. Scand J Work Environ Health. (2004) 30:149–56. doi: 10.5271/sjweh.772, PMID: 15143742

[58] Di Muzio M Diella G Di Simone E Pazzaglia M Alfonsi V Novelli L. Comparison of sleep and attention metrics among nurses working shifts on a forward-vs backward-rotating schedule. JAMA Netw Open. (2021) 4:e2129906. doi: 10.1001/jamanetworkopen.2021.29906 34661660 PMC8524311

[59] Statbel (2023) Available at: https://statbel.fgov.be/nl/themas/werk-opleiding/opleidingen-en-onderwijs/onderwijsniveau#figures (accessed May 2023).

[60] Baranowski M Jabkowski P. Gender and socioeconomic patterning of self-reported sleep problems across European countries. Eur J Pub Health. (2023) 33:242–8. doi: 10.1093/eurpub/ckad012, PMID: 36805658 PMC10066480

[61] Hornberger S Knauth P. Effects of various types of change in shift schedules: a controlled longitudinal study. Work Stress. (1995) 9:124–33. doi: 10.1080/02678379508256546

[62] Kecklund G Eriksen CA Åkerstedt T. Police officers attitude to different shift systems: association with age, present shift schedule, health and sleep/wake complaints. Appl Ergon. (2008) 39:565–71. doi: 10.1016/j.apergo.2008.01.002, PMID: 18281011

