# Supplementary material for: Shift work is associated with extensively disordered sleep, especially when working nights
Source: Front Psychiatry. 2023 Dec 7;14:1233640. doi: 10.3389/fpsyt.2023.1233640 (PMC10755475; doi:10.3389/fpsyt.2023.1233640)
Supplement: Supplementary file 1 [file Table_1.docx]

Supplement 1: Statistical analyses of prevalence of short sleep duration and sleep disorders in relation to different demographic factors.

TABLE S1.1 Prevalence’s ratio’s for the comparison of demographic factors with the reference (indicator) variable.

|  | **<7hrs sleep** | **≥1 sleep disorders** | **≥2 sleep disorders** | **Insomnia** | **SBD** | **Hypersomnia** | **Parasomnia** | **CRSWD** | **SRMD** |
| --- | --- | --- | --- | --- | --- | --- | --- | --- | --- |
| ***Sex*** | | | | | | | | | |
| *males*  *(n=* *15279)* | 1 | 1 | 1 | 1 | 1 | 1 | 1 | 1 | 1 |
| *females*  *(n=* *22383)* | 0.81 **  (0.77-0.86) | 1.16 **  (1.12-1.21) | 1.25 **  (1.19-1.32) | 1.45 **  (1.38-1.51) | 0.52 **  (0.43-0.60) | 1.54 **  (1.45-1.63) | 1.85 **  (1.75-1.96) | 1.13 *  (1.05-1.20) | 1.17*  (1.10-1.23) |
| ***Age*** | | | | | | | | | |
| *< 30 (n=9146)* | 1 | 1 | 1 | 1 | 1 | 1 | 1 | 1 | 1 |
| *30-50 (n=18450)* | 1.32 **  (1.26-1.38) | 0.80 **  (0.75-0.85) | 0.64 **  (0.56-0.71) | 0.82 **  (0.75-0.89) | 1.67 **  (1.54-1.80) | 0.6 **  (0.50-0.70) | 0.49 **  (0.39-0.59) | 0.53 **  (0.45-0.61) | 0.87*  (0.79-0.94) |
| *> 50 (n=10066)* | 1.79 **  (1.73-1.86) | 0.83 **  (0.77-0.89) | 0.74 **  (0.66-0.82) | 0.75 **  (0.67-0.84) | 2.76 **  (2.62-2.89) | 0.63 **  (0.52-0.74) | 0.31 **  (0.17-0.45) | 0.56 **  (0.46-0.65) | 1.04  (0.96-1.13) |
| ***Education*** | | | | | | | | | |
| *academic (n=22119)* | 1 | 1 | 1 | 1 | 1 | 1 | 1 | 1 | 1 |
| *vocational (n=9000)* | 1.25 **  (1.19-1.30) | 1.17*  (1.12-1.22) | 1.31**  (1.24-1.39) | 1.23 **  (1.16-1.31) | 1.35 **  (1.25-1.46) | 1.33 **  (1.23-1.43) | 1.02  (0.91-1.13) | 1.19 **  (1.11-1.28) | 1.23 **  (1.16-1.31) |
| *secondary (n=6401)* | 1.56 V  (1.50-1.62) | 1.45 **  (1.40-1.51) | 1.94 **  (1.87-2.02) | 1.52 **  (1.44-1.60) | 1.83 **  (1.73-1.94) | 1.98 **  (1.88-2.08) | 1.42 **  (1.30-1.53) | 1.87 **  (1.79-1.96) | 1.65 **  (1.57-1.72) |
| *elementary (n=142)* | 1.96 **  (1.63-2.29) | 1.78 **  (1.45-2.11) | 2.21 **  (1.81-2.60) | 1.63 **  (1.21-2.06) | 3.23 **  (2.77-3.69) | 2.08 **  (1.54-2.62) | 1.77 **  (1.18-2.36) | 2.35 **  (1.92-2.77) | 2.08 **  (1.69-2.48) |
| ***Living companion(s)*** | | | | | | | | | |
| *alone (n=4705)* | 1 | 1 | 1 | 1 | 1 | 1 | 1 | 1 | 1 |
| *with partner (n=10663)* | 0.72 **  (0.64-0.80) | 0.96  (0.88-1.03) | 0.81 **  (0.71-0.90) | 0.78 **  (0.68-0.88) | 1.02  (0.87-1.16) | 0.80 *  (0.66-0.94) | 1.02  (0.87-1.16) | 0.66 **  (0.55-0.77) | 1.15 *  (1.04-1.25) |
| *alone with kids (n=2041)* | 1.07  (0.96-1.18) | 0.94  (0.83-1.05) | 0.79 **  (0.63-0.94) | 0.89  (0.74-1.04) | 1.10  (0.89-1.30) | 1.04  (0.84-1.24) | 0.73 *  (0.48-0.97) | 0.59 **  (0.41-0.77) | 0.98  (0.82-1.14) |
| *with partner and kids (n=14895)* | 0.85 **  (0.78-0.92) | 0.81 **  (0.74-0.88) | 0.63 **  (0.54-0.73) | 0.75 **  (0.65-0.84) | 0.95  (0.82-1.09) | 0.79 *  (0.65-0.92) | 0.54 **  (0.39-0.69) | 0.44 **  (0.34-0.55) | 0.93  (0.83-1.04) |
| *other (n=5280)* | 0.77 **  (0.68-0.85) | 1.12 *  (1.04-1.20) | 1,20 *  (1.09-1.30) | 1.11 *  (1.00-1.22) | 0.65 **  (0.47-0.83) | 1.36 **  (1.21-1.50) | 1.44 **  (1.29-1.60) | 1.22 **  (1.11-1.33) | 1.11 *  (1.00-1.23) |
| ***Work schedule*** | | | | | | | | | |
| *day (n=32468)* | 1 | 1 | 1 | 1 | 1 | 1 | 1 | 1 | 1 |
| *early (n=434)* | 1.72 **  (1.53-1.91) | 1.31 **  (1.12-1.50) | 1.66 **  (1.42-1.90) | 1.35*  (1.09-1.60) | 1.77 **  (1.45-2.09) | 2.02 **  (1.71-2.32) | 1.51 **  (1.15-1.87) | 1.42 **  (1.12-1.73) | 1.21  (0.95-1.48) |
| *evening (n=507)* | 0.95  (0.75-1.15) | 1.36 **  (1.18-1.54) | 1.74 **  (1.51-1.95) | 1.41 **  (1.18-1.65) | 1.20  (0.85-1.55) | 1.73 **  (1.43-2.03) | 1.49 **  (1.15-1.83) | 2.58 **  (2.36-2.80) | 1.21  (0.96-1.46) |
| *night (n=186)* | 1.88 **  (1.59-2.17) | 1.62 **  (1.33-1.91) | 2.32 **  (1.99-2.64) | 1.62 **  (1.26-1.98) | 1.82 **  (1.34-2.30) | 2.84 **  (2.44-3.24) | 1.65 **  (1.12-2.18) | 3.17 **  (2.83-3.50) | 1.87 **  (1.52-2.21) |
| *rotating (n=4067)* | 1.29 **  (1.22-1.36) | 1.41 **  (1.35-1.48) | 1.81 **  (1.72-1.89) | 1.25 **  (1.16-1.34) | 1.34 **  (1.21-1.47) | 2.20 **  (2.09-2.31) | 1.41 **  (1.28-1.54) | 2.49 **  (2.40-2.57) | 1.31 **  (1.22-1.40) |

CRSWD = circadian rhythm sleep-wake disorder, SBD = sleep breathing disorder, SRMD = sleep-related movement disorder. * p<0.05, ** P<0.01

Supplement 2: Summary of statistics of binary logistic analyses in day, early, evening, night and rotation shift subgroups.

TABLE S2.1 Binary logistic regression to assess associations between short sleep duration and sex, age, education level and living companion in day, evening, night and rotation shift subgroups.

|  | **B** | **S.E.** | **Wald** | **df** | **Sig.** | **Exp(B)** | **95% C.I.for EXP(B)** | |
| --- | --- | --- | --- | --- | --- | --- | --- | --- |
| **Day shift (Cox & Snell R Square = 0.032; p<0.001)** | | | | | | | | |
| **Sex** *(Male vs Female)* | -0.208 | 0.219 | 1 | 1 | 0.034 | 0.812 | 0.529 | 1.247 |
| **Age** |  |  | 7 | 2 | 0.026 |  |  |  |
| *30-50 vs <30* | -0.109 | 0.326 | 0 | 1 | 0.739 | 1.000 | 0.474 | 1.698 |
| *>50 vs <30* | 0.544 | 0.334 | 3 | 1 | 0.104 | 2.000 | 0.895 | 3.315 |
| **Education** |  |  | 269 | 3 | <0.001 |  |  |  |
| *elementary vs academic* | 0.831 | 0.207 | 16 | 1 | <0.001 | 2.000 | 1.530 | 3.448 |
| *secondary vs academic* | 0.557 | 0.036 | 246 | 1 | <0.001 | 2.000 | 1.628 | 1.871 |
| *vocational vs academic* | 0.238 | 0.031 | 60 | 1 | <0.001 | 1.269 | 1.194 | 1.348 |
| **Living companion(s)** |  |  | 155 | 4 | <0.001 |  |  |  |
| *with partner vs alone* | -0.452 | 0.043 | 109 | 1 | <0.001 | 0.636 | 0.584 | 0.692 |
| *alone with kids vs alone* | 0.009 | 0.062 | 0.019 | 1 | 0.890 | 1.009 | 0.893 | 1.138 |
| *with partner and kids vs alone* | -0.267 | 0.041 | 43 | 1 | <0.001 | 0.765 | 0.707 | 0.829 |
| *other vs alone* | -0.106 | 0.055 | 3.68 | 1 | 0.055 | 0.899 | 0.807 | 1.002 |
| **Constant** | -1.000 | 0.052 | 568.768 | 1 | <0.001 | 0.292 |  |  |
| **Early shift (Cox & Snell R Square = 0.082; p<0.001)** | | | | | | | | |
| **Sex** *(Male vs Female)* | -0.451 | 0.204 | 4.882 | 1 | 0.027 | 0.637 | 0.427 | 0.950 |
| **Age** |  |  | 6.796 | 2 | 0.033 |  |  |  |
| *30-50 vs <30* | 0.345 | 0.334 | 1.062 | 1 | 0.303 | 1.412 | 0.733 | 2.719 |
| *>50 vs <30* | 0.798 | 0.334 | 5.696 | 1 | 0.017 | 2.221 | 1.153 | 4.276 |
| **Education** |  |  | 20.344 | 3 | 0 |  |  |  |
| *elementary vs academic* | 1.069 | 0.723 | 2.186 | 1 | 0.139 | 2.913 | 0.706 | 12.022 |
| *secondary vs academic* | 1.031 | 0.245 | 17.677 | 1 | 0 | 2.805 | 1.734 | 4.536 |
| *vocational vs academic* | 0.262 | 0.296 | 0.783 | 1 | 0.376 | 1.299 | 0.728 | 2.320 |
| **Living companion(s)** |  |  | 3.393 | 4 | 0.494 |  |  |  |
| *with partner vs alone* | -0.102 | 0.325 | 0.099 | 1 | 0.753 | 0.903 | 0.477 | 1.708 |
| *alone with kids vs alone* | 0.368 | 0.509 | 0.523 | 1 | 0.47 | 1.445 | 0.533 | 3.921 |
| *with partner and kids vs alone* | 0.217 | 0.313 | 0.481 | 1 | 0.488 | 1.242 | 0.673 | 2.294 |
| *other vs alone* | 0.473 | 0.402 | 1.384 | 1 | 0.239 | 1.605 | 0.730 | 3.531 |
| **Constant** | -1.107 | 0.408 | 7.371 | 1 | 0.007 | 0.331 |  |  |
| **Evening shift (Cox & Snell R Square = 0.047; p=0.006)** | | | | | | | | |
| **Sex** *(Male vs Female)* | -0.208 | 0.219 | 0.906 | 1 | 0.341 | 0.812 | 0.529 | 1.247 |
| **Age** |  |  | 7 | 2 | 0.026 |  |  |  |
| *30-50 vs <30* | -0.109 | 0.326 | 0.111 | 1 | 0.739 | 0.897 | 0.474 | 1.698 |
| *>50 vs <30* | 0.544 | 0.334 | 3 | 1 | 0.104 | 2.000 | 0.895 | 3.315 |
| **Education** |  |  | 8 | 3 | 0.04 |  |  |  |
| *elementary vs academic** | 2.565 | 1.14 | 5.059 | 1 | 0.025 | 12.996 | 1.391 | 121.451 |
| *secondary vs academic* | 0.343 | 0.254 | 2 | 1 | 0.177 | 1.000 | 0.857 | 2.318 |
| *vocational vs academic* | -0.267 | 0.303 | 0.772 | 1 | 0.379 | 0.766 | 0.423 | 1.388 |
| **Living companion(s)** |  |  | 4 | 4 | 0.355 |  |  |  |
| *with partner vs alone* | -0.283 | 0.33 | 0.734 | 1 | 0.392 | 0.754 | 0.395 | 1.439 |
| *alone with kids vs alone* | 0.608 | 0.48 | 2 | 1 | 0.205 | 2.000 | 0.717 | 4.707 |
| *with partner and kids vs alone* | 0.123 | 0.318 | 0.15 | 1 | 0.699 | 1.000 | 0.606 | 2.110 |
| *other vs alone* | -0.282 | 0.39 | 0.523 | 1 | 0.47 | 0.754 | 0.351 | 1.621 |
| **Constant** | -1 | 0.384 | 8 | 1 | 0.004 | 0.327 |  |  |
| **Night shift (Cox & Snell R Square = 0.081; p=0.08)** | | | | | | | |  |
| **Sex** *(Male vs Female)* | -0.498 | 0.364 | 2 | 1 | 0.172 | 0.608 | 0.298 | 1.241 |
| **Age** |  |  | 4 | 2 | 0.168 |  |  |  |
| *30-50 vs <30* | 0.711 | 0.591 | 1 | 1 | 0.229 | 2.035 | 0.639 | 6.481 |
| *>50 vs <30* | 1.173 | 0.639 | 3 | 1 | 0.066 | 3.233 | 0.923 | 11.318 |
| **Education** |  |  | 8 | 2 | 0.018 |  |  |  |
| *elementary vs academic** |  |  |  |  |  |  |  |  |
| *secondary vs academic* | 1.244 | 0.446 | 7.787 | 1 | 0.005 | 3.471 | 1.448 | 8.319 |
| *vocational vs academic* | 0.000 | 0.427 | 1 | 1 | 0.277 | 1.592 | 0.689 | 3.680 |
| **Living companion(s)** |  |  | 8 | 3 | 0.04 |  |  |  |
| *with partner vs alone* | 2.565 | 1.14 | 5.059 | 1 | 0.025 | 12.996 | 1.391 | 121.451 |
| *alone with kids vs alone** |  |  |  |  |  |  |  |  |
| *with partner and kids vs alone* | 0.322 | 0.491 | 0.431 | 1 | 0.512 | 1.380 | 0.527 | 3.613 |
| *other vs alone* | 0.414 | 0.677 | 0.374 | 1 | 0.541 | 1.513 | 0.401 | 5.704 |
| **Constant** | -1.319 | 0.771 | 3 | 1 | 0.087 | 0.267 | - |  |
| **Rotating shifts (Cox & Snell R Square = 0.044; p<0.001)** | | | | | | | |  |
| **Sex** *(Male vs Female)* | -0.311 | 0.069 | 20 | 1 | 0 | 0.733 | 0.640 | 0.839 |
| **Age** |  |  | 61 | 2 | 0 |  |  |  |
| *30-50 vs <30* | 0.313 | 0.099 | 10 | 1 | 0.002 | 1.000 | 1.126 | 1.662 |
| *>50 vs <30* | 0.783 | 0.105 | 55 | 1 | 0 | 2.000 | 1.780 | 2.688 |
| **Education** |  |  | 35 | 3 | 0 |  |  |  |
| *elementary vs academic* | 0.767 | 0.41 | 3.49 | 1 | 0.062 | 2.000 | 0.963 | 4.812 |
| *secondary vs academic* | 0.456 | 0.08 | 33 | 1 | 0 | 2.000 | 1.349 | 1.845 |
| *vocational vs academic* | 0.212 | 0.085 | 6 | 1 | 0.013 | 1.000 | 1.045 | 1.461 |
| **Living companion(s)** |  |  | 43 | 4 | 0 |  |  |  |
| *with partner vs alone* | -0.619 | 0.11 | 31.479 | 1 | 0 | 0.539 | 0.434 | 0.669 |
| *alone with kids vs alone* | 0.053 | 0.176 | 0.09 | 1 | 0.765 | 1.000 | 0.746 | 1.490 |
| *with partner and kids vs alone* | -0.190 | 0.106 | 3 | 1 | 0.074 | 0.827 | 0.671 | 1.018 |
| *other vs alone* | -0.171 | 0.124 | 2 | 1 | 0.167 | 0.843 | 0.662 | 1.074 |
| **Constant** | -0.774 | 0.123 | 39 | 1 | 0 | 0.461 |  |  |

TABLE S2.2 Binary logistic regression to assess associations between the prevalence of at least one sleep disorder and sex, age, education level and living companion in day, early, evening, night and rotation shift subgroups.

|  | **B** | **S.E.** | **Wald** | **df** | **Sig.** | **Exp(B)** | **95% C.I.for EXP(B)** | |
| --- | --- | --- | --- | --- | --- | --- | --- | --- |
| **Day shift (Cox & Snell R Square = 0.017; p<0.001)** | | | | | | | | |
| **Sex** *(Male vs Female)* | 0.224 | 0.025 | 81 | 1 | <0.001 | 1.000 | 1.192 | 1.314 |
| **Age** |  |  | 23 | 2 | <0.001 |  |  |  |
| *30-50 vs <30* | -0.140 | 0.037 | 15 | 1 | <0.001 | 0.869 | 0.809 | 0.934 |
| *>50 vs <30* | -0.185 | 0.039 | 23 | 1 | <0.001 | 0.832 | 0.771 | 0.897 |
| **Education** |  |  | 277.49 | 3 | <0.001 |  |  |  |
| *elementary vs academic* | 0.837 | 0.203 | 17 | 1 | <0.001 | 2.000 | 1.553 | 3.437 |
| *secondary vs academic* | 0.537 | 0.034 | 251.371 | 1 | <0.001 | 1.710 | 1.600 | 1.827 |
| *vocational vs academic* | 0.229 | 0.029 | 61 | 1 | <0.001 | 1.000 | 1.188 | 1.332 |
| **Living companion(s)** |  |  | 60 | 4 | <0.001 |  |  |  |
| *with partner vs alone* | -0.021 | 0.041 | 0.264 | 1 | 0.608 | 0.979 | 0.904 | 1.061 |
| *alone with kids vs alone* | -0.045 | 0.062 | 0.542 | 1 | 0.462 | 0.956 | 0.847 | 1.078 |
| *with partner and kids vs alone* | -0.214 | 0.040 | 29 | 1 | <0.001 | 0.807 | 0.746 | 0.873 |
| *other vs alone* | 0.066 | 0.050 | 2 | 1 | 0.191 | 1.000 | 0.968 | 1.179 |
| **Constant** | -0.853 | 0.047 | 330.41 | 1 | <0.001 | 0.426 |  |  |
| **Early shift (Cox & Snell R Square = 0.026; p<0.001)** | | | | | | | | |
| **Sex** *(Male vs Female)* | -0.096 | 0.200 | 0.228 | 1 | 0.633 | 0.909 | 0.614 | 1.346 |
| **Age** |  |  | 0.769 | 2 | 0.681 |  |  |  |
| *30-50 vs <30* | -0.064 | 0.317 | 0.04 | 1 | 0.841 | 0.938 | 0.504 | 1.747 |
| *>50 vs <30* | -0.236 | 0.316 | 0.557 | 1 | 0.456 | 0.790 | 0.425 | 1.467 |
| **Education** |  |  | 8.414 | 3 | 0.038 |  |  |  |
| *elementary vs academic* | 1.263 | 0.738 | 2.933 | 1 | 0.087 | 3.536 | 0.833 | 15.007 |
| *secondary vs academic* | 0.453 | 0.236 | 3.682 | 1 | 0.055 | 1.573 | 0.990 | 2.498 |
| *vocational vs academic* | -0.135 | 0.294 | 0.211 | 1 | 0.646 | 0.874 | 0.491 | 1.554 |
| **Living companion(s)** |  |  | 1.732 | 4 | 0.785 |  |  |  |
| *with partner vs alone* | 0.420 | 0.322 | 1.697 | 1 | 0.193 | 1.522 | 0.809 | 2.864 |
| *alone with kids vs alone* | 0.246 | 0.504 | 0.238 | 1 | 0.625 | 1.279 | 0.476 | 3.438 |
| *with partner and kids vs alone* | 0.250 | 0.314 | 0.637 | 1 | 0.425 | 1.285 | 0.694 | 2.377 |
| *other vs alone* | 0.194 | 0.389 | 0.251 | 1 | 0.617 | 1.215 | 0.567 | 2.601 |
| **Constant** | -0.663 | 0.389 | 2.909 | 1 | 0.088 | 0.515 |  |  |
| **Evening shifts (Cox & Snell R Square = 0.052; p=0.003)** | | | | | | | | |
| **Sex** *(Male vs Female)* | 0.053 | 0.194 | 0.074 | 1 | 0.785 | 1.000 | 0.721 | 1.541 |
| **Age** |  |  | 5 | 2 | 0.069 |  |  |  |
| *30-50 vs <30* | -0.174 | 0.268 | 0.424 | 1 | 0.515 | 0.840 | 0.497 | 1.420 |
| *>50 vs <30* | -0.614 | 0.294 | 4 | 1 | 0.037 | 0.541 | 0.304 | 0.962 |
| **Education** |  |  | 9 | 3 | 0.027 |  |  |  |
| *elementary vs academic** |  |  |  |  |  |  |  |  |
| *secondary vs academic* | 0.489 | 0.228 | 5 | 1 | 0.032 | 2.000 | 1.043 | 2.548 |
| *vocational vs academic* | 0.594 | 0.250 | 6 | 1 | 0.018 | 2.000 | 1.109 | 2.957 |
| **Living companion(s)** |  |  | 7 | 4 | 0.155 |  |  |  |
| *with partner vs alone* | -0.311 | 0.280 | 1 | 1 | 0.266 | 0.733 | 0.423 | 1.268 |
| *alone with kids vs alone* | -0.092 | 0.459 | 0.04 | 1 | 0.842 | 0.912 | 0.371 | 2.244 |
| *with partner and kids vs alone* | -0.69 | 0.285 | 6 | 1 | 0.015 | 0.502 | 0.287 | 0.876 |
| *other vs alone* | -0.176 | 0.325 | 0.295 | 1 | 0.587 | 0.838 | 0.443 | 1.585 |
| **Constant** | 0.011 | 0.328 | 0.001 | 1 | 0.973 | 1.000 |  |  |
| **Night shift (Cox & Snell R Square = 0.048; p=0.413)** | | | | | | | | |
| **Sex** *(Male vs Female)* | 0.666 | 0.361 | 3 | 1 | 0.065 | 1.947 | 0.960 | 3.95 |
| **Age** |  |  | 0.671 | 2 | 0.715 |  |  |  |
| *30-50 vs <30* | -0.412 | 0.569 | 0.525 | 1 | 0.469 | 0.662 | 0.217 | 2.020 |
| *>50 vs <30* | -0.205 | 0.609 | 0.113 | 1 | 0.737 | 0.815 | 0.247 | 2.688 |
| **Education** |  |  | 0.843 | 2 | 0.656 |  |  |  |
| *elementary vs academic** |  |  |  |  |  |  |  |  |
| *secondary vs academic* | 0.051 | 0.428 | 0.014 | 1 | 0.906 | 1.052 | 0.455 | 2.436 |
| *vocational vs academic* | 0.346 | 0.417 | 0.689 | 1 | 0.406 | 1.413 | 0.625 | 3.197 |
| **Constant** | 0.288 | 0.737 | 0.153 | 1 | 0.696 | 1.334 |  |  |
| *with partner vs alone* | -0.834 | 0.531 | 2 | 1 | 0.116 | 0.434 | 0.153 | 1.230 |
| *alone with kids vs alone** |  |  |  |  |  |  |  |  |
| *with partner and kids vs alone* | -0.722 | 0.489 | 2 | 1 | 0.140 | 0.486 | 0.186 | 1.268 |
| *other vs alone* | -0.007 | 0.661 | 0 | 1 | 0.991 | 0.993 | 0.272 | 3.628 |
| **Constant** | 0.288 | 0.737 | 0.153 | 1 | 0.696 | 1.334 |  |  |
| **Rotating shifts (Cox & Snell R Square = 0.018; p<0.001)** | | | | | | | | |
| **Sex** *(Male vs Female)* | 0.162 | 0.066 | 6 | 1 | 0.013 | 1.000 | 1.035 | 1.337 |
| **Age** |  |  | 6 | 2 | 0.049 |  |  |  |
| *30-50 vs <30* | -0.217 | 0.090 | 6 | 1 | 0.016 | 0.805 | 0.675 | 0.960 |
| *>50 vs <30* | -0.178 | 0.097 | 3 | 1 | 0.065 | 0.837 | 0.693 | 1.011 |
| **Education** |  |  | 26 | 3 | <0.001 |  |  |  |
| *elementary vs academic* | 0.663 | 0.409 | 2.624 | 1 | 0.105 | 2.000 | 0.870 | 4.325 |
| *secondary vs academic* | 0.363 | 0.076 | 23 | 1 | <0.001 | 1.000 | 1.240 | 1.668 |
| *vocational vs academic* | 0.226 | 0.080 | 8 | 1 | 0.005 | 1.000 | 1.072 | 1.467 |
| **Living companion(s)** |  |  | 20 | 4 | 0.001 |  |  |  |
| *with partner vs alone* | -0.333 | 0.103 | 10 | 1 | 0.001 | 0.717 | 0.585 | 0.878 |
| *alone with kids vs alone* | -0.300 | 0.175 | 3 | 1 | 0.086 | 0.741 | 0.526 | 1.043 |
| *with partner and kids vs alone* | -0.406 | 0.103 | 15 | 1 | <0.001 | 0.667 | 0.544 | 0.816 |
| *other vs alone* | -0.106 | 0.116 | 0.836 | 1 | 0.361 | 0.899 | 0.716 | 1.129 |
| **Constant** | -0.097 | 0.115 | 0.716 | 1 | 0.397 | 0.907 |  |  |

TABLE S2.3 Binary logistic regression to assess associations between the prevalence of CRSWD and sex, age, education level and living companion in day, early, evening, night and rotation shift subgroups.

|  | **B** | **S.E.** | **Wald** | **df** | **Sig.** | **Exp(B)** | **95% C.I.for EXP(B)** | |
| --- | --- | --- | --- | --- | --- | --- | --- | --- |
| **Day shift (Cox & Snell R Square = 0.017; p<0.001)** | | | | | | | |  |
| **Sex** *(Male vs Female)* | 0.170 | 0.044 | 15 | 1 | <0.001 | 1.000 | 1.088 | 1.291 |
| **Age** |  |  | 36 | 2 | <0.001 |  |  |  |
| *30-50 vs <30* | -0.278 | 0.060 | 22 | 1 | <0.001 | 0.757 | 0.674 | 0.851 |
| *>50 vs <30* | -0.380 | 0.065 | 34 | 1 | <0.001 | 0.684 | 0.602 | 0.776 |
| **Education** |  |  | 118 | 3 | <0.001 |  |  |  |
| *elementary vs academic* | 0.890 | 0.285 | 10 | 1 | 0.002 | 2.000 | 1.393 | 4.255 |
| *secondary vs academic* | 0.566 | 0.054 | 111 | 1 | <0.001 | 2.000 | 1.585 | 1.956 |
| *vocational vs academic* | 0.203 | 0.052 | 15 | 1 | <0.001 | 1.000 | 1.106 | 1.358 |
| **Living companion(s)** |  |  | 196 | 4 | <0.001 |  |  |  |
| *with partner vs alone* | -0.452 | 0.065 | 49 | 1 | <0.001 | 0.636 | 0.561 | 0.722 |
| *alone with kids vs alone* | -0.448 | 0.105 | 18 | 1 | <0.001 | 0.639 | 0.52 | 0.785 |
| *with partner and kids vs alone* | -0.800 | 0.066 | 148 | 1 | <0.001 | 0.450 | 0.395 | 0.511 |
| *other vs alone* | 0.016 | 0.074 | 0.049 | 1 | 0.825 | 1.000 | 0.879 | 1.176 |
| **Constant** | -2.080 | 0.073 | 811 | 1 | <0.001 | 0.125 |  |  |
| **Early shift (Cox & Snell R Square = 0.07; p<0.001)** | | | | | | | |  |
| **Sex** *(Male vs Female)* | 0.370 | 0.328 | 1.276 | 1 | 0.259 | 1.448 | 0.762 | 2.754 |
| **Age** |  |  | 4.982 | 2 | 0.083 |  |  |  |
| *30-50 vs <30* | -0.365 | 0.550 | 0.442 | 1 | 0.506 | 0.694 | 0.236 | 2.038 |
| *>50 vs <30* | 0.606 | 0.489 | 1.537 | 1 | 0.215 | 1.834 | 0.703 | 4.784 |
| **Education** |  |  | 4.885 | 3 | 0.180 |  |  |  |
| *elementary vs academic** | 1.144 | 0.925 | 1.532 | 1 | 0.216 | 3.140 | 0.513 | 19.229 |
| *secondary vs academic* | 0.375 | 0.388 | 0.936 | 1 | 0.333 | 1.456 | 0.680 | 3.114 |
| *vocational vs academic* | -0.652 | 0.619 | 1.109 | 1 | 0.292 | 0.521 | 0.155 | 1.754 |
| **Living companion(s)** |  |  | 10.046 | 4 | 0.040 |  |  |  |
| *with partner vs alone* | -0.025 | 0.589 | 0.002 | 1 | 0.966 | 0.975 | 0.308 | 3.092 |
| *alone with kids vs alone* | 0.151 | 0.889 | 0.029 | 1 | 0.865 | 1.164 | 0.204 | 6.640 |
| *with partner and kids vs alone* | 0.128 | 0.586 | 0.048 | 1 | 0.827 | 1.137 | 0.360 | 3.586 |
| *other vs alone* | 1.503 | 0.611 | 6.046 | 1 | 0.014 | 4.493 | 1.356 | 14.883 |
| **Constant** | -3.011 | 0.688 | 19.156 | 1 | 0.000 | 0.049 |  |  |
| **Evening shift (Cox & Snell R Square = 0.066; p<0.001)** | | | | | | | |  |
| **Sex** *(Male vs Female)* | -0.248 | 0.238 | 1 | 1 | 0.296 | 0.780 | 0.490 | 1.243 |
| **Age** |  |  | 7 | 2 | 0.025 |  |  |  |
| *30-50 vs <30* | -0.395 | 0.311 | 2 | 1 | 0.204 | 0.674 | 0.366 | 1.239 |
| *>50 vs <30* | -1.000 | 0.375 | 7 | 1 | 0.007 | 0.363 | 0.174 | 0.758 |
| **Education** |  |  | 6 | 3 | 0.131 |  |  |  |
| *elementary vs academic** |  |  |  |  |  |  |  |  |
| *secondary vs academic* | 0.442 | 0.272 | 3 | 1 | 0.104 | 2.000 | 0.913 | 2.653 |
| *vocational vs academic* | 0.380 | 0.314 | 1 | 1 | 0.226 | 1 | 0.790 | 2.703 |
| **Living companion(s)** |  |  | 11 | 4 | 0.026 |  |  |  |
| *with partner vs alone* | -0.698 | 0.331 | 4 | 1 | 0.035 | 0.497 | 0.260 | 0.952 |
| *alone with kids vs alone* | -0.294 | 0.566 | 0.269 | 1 | 0.604 | 0.746 | 0.246 | 2.263 |
| *with partner and kids vs alone* | -1.160 | 0.371 | 10 | 1 | 0.002 | 0.314 | 0.152 | 0.648 |
| *other vs alone* | -0.351 | 0.361 | 0.948 | 1 | 0.330 | 0.704 | 0.347 | 1.427 |
| **Constant** | -0.477 | 0.372 | 2 | 1 | 0.200 | 0.621 |  |  |
| **Night shift (Cox & Snell R Square = 0.060; p=0.243)** | | | | | | | |  |
| **Sex** *(Male vs Female)* | 0.536 | 0.427 | 2 | 1 | 0.210 | 1.709 | 0.740 | 3.948 |
| **Age** |  |  | 3 | 2 | 0.227 |  |  |  |
| *30-50 vs <30* | -0.410 | 0.670 | 0.375 | 1 | 0.540 | 0.664 | 0.179 | 2.466 |
| *>50 vs <30* | 0.351 | 0.725 | 0.235 | 1 | 0.628 | 1.421 | 0.343 | 5.886 |
| **Education** |  |  | 0.431 | 2 | 0.806 |  |  |  |
| *elementary vs academic** |  |  |  |  |  |  | - |  |
| *secondary vs academic* | -0.059 | 0.499 | 0.014 | 1 | 0.906 | 0.943 | 0.355 | 2.506 |
| *vocational vs academic* | 0.236 | 0.482 | 0.239 | 1 | 0.625 | 1.266 | 0.492 | 3.259 |
| **Living companion(s)** |  |  | 7 | 3 | 0.084 |  |  |  |
| *with partner vs alone* | -1.118 | 0.614 | 3 | 1 | 0.069 | 0.327 | 0.098 | 1.089 |
| *alone with kids vs alone** |  |  |  |  |  |  |  |  |
| *with partner and kids vs alone* | -0.360 | 0.536 | 0.45 | 1 | 0.502 | 0.698 | 0.244 | 1.997 |
| *other vs alone* | 0.640 | 0.724 | 0.782 | 1 | 0.377 | 1.896 | 0.459 | 7.831 |
| **Constant** | -1.170 | 0.864 | 2 | 1 | 0.176 | 0.310 | - |  |
| **Rotating shifts (Cox & Snell R Square = 0.024; p<0.001)** | | | | | | | |  |
| **Sex** *(Male vs Female)* | -0.045 | 0.082 | 0.295 | 1 | 0.587 | 0.956 | 0.814 | 1.124 |
| **Age** |  |  | 10 | 2 | 0.008 |  |  |  |
| *30-50 vs <30* | -0.314 | 0.110 | 8 | 1 | 0.004 | 0.731 | 0.589 | 0.907 |
| *>50 vs <30* | -0.316 | 0.120 | 7 | 1 | 0.009 | 0.729 | 0.576 | 0.923 |
| **Education** |  |  | 15 | 3 | 0.002 |  |  |  |
| *elementary vs academic* | 0.258 | 0.510 | 0.256 | 1 | 0.613 | 1.000 | 0.476 | 3.517 |
| *secondary vs academic* | 0.337 | 0.095 | 13 | 1 | <0.001 | 1.000 | 1.163 | 1.686 |
| *vocational vs academic* | 0.295 | 0.102 | 8 | 1 | 0.004 | 1.000 | 1.100 | 1.640 |
| **Living companion(s)** |  |  | 41 | 4 | <0.001 |  |  |  |
| *with partner vs alone* | -0.466 | 0.123 | 14 | 1 | <0.001 | 0.627 | 0.493 | 0.798 |
| *alone with kids vs alone* | -0.761 | 0.242 | 10 | 1 | 0.002 | 0.467 | 0.291 | 0.750 |
| *with partner and kids vs alone* | -0.744 | 0.128 | 34 | 1 | <0.001 | 0.475 | 0.370 | 0.611 |
| *other vs alone* | -0.152 | 0.133 | 1 | 1 | 0.253 | 0.859 | 0.662 | 1.115 |
| **Constant** | -0.98 | 0.136 | 52.225 | 1 | <0.001 | 0.375 |  |  |
